# Supplementary material for: PTBP1 mediates Sertoli cell actin cytoskeleton organization by regulating alternative splicing of actin regulators
Source: Nucleic Acids Res. 2024 Oct 7;52(20):12244–61. doi: 10.1093/nar/gkae862 (PMC11551747; doi:10.1093/nar/gkae862)
Supplement: gkae862_Supplemental_Files [file gkae862_supplemental_files.zip › Supplementary Tables 8.27.24.pdf]

Supplementary Table 1. Primer sequences for genotyping

Genes	Forward (5’ to 3’)	Reverse (5’ to 3’)
Ptbp1 floxed allele	CCCATAACTGTCCATAGACC	TGTTGGTAATGCCAGCACAG
Amh-cre	GTGTGGGACAGAGAACAAACCG	TGCGAACCTCATCACTCGTTGC

Supplementary Table 2. Primer sequences for the splicing assay

Genes	Forward (5’ to 3’)	Reverse (5’ to 3’)
*Tnik*_exon14	AAGAACAGATGCGTCGGGAG	GGGAAACCAGATAGTCCCGC
*Tnik*_exon21	CCATGAGCCTTCCAAGGTGAA	CTTTCTTCAGGGGGCGGTT
*Espn*_exon11	AGCGTCGTTCCTCGTCTT	TCTCAGCCAGAAGCTCTGAG
*Ppp1r9a*_exon14	AGTCCAAGCAGTACCAGCTC	TAACCAGTGGGACACCTGCT
*Rab11fip3*_exon6	GGCAATGAGGCAGAACTATC	CTTGCCACCTTCCTGCTGGA
*Ppfibp2*_exon16	AAGCTGCCAAATCTCCTCCC	CTTCCCAGCCTCCTTCTGTG
*Kif21a*_exon29	GCCAGAGCCTTCCCCTGTAA	CACCTCGGACAAAGAGGAGT
*Camkk2*_exon14	TCCAGAGTCCAGGATTGTGGT	CGAATCATGGTCTTTACCAGGA

Supplementary Table 3. Primer sequences for quantitative PCR

Genes	Forward (5’ to 3’)	Reverse (5’ to 3’)
Tardbp	ATGAGAACGATGAACCCATTGAA	TGAGACACGGGATTCCGGTAG
Actb	GGCTGTATTCCCCTCCATCG	CCAGTTGGTAACAATGCCATGT
Actg1	AATCGCCGCACTCGTCATT	CCCTACGATGGAAGGGAACAC
Gapdh	TGCACCACCAACTGCTTAGC	GGCATGGACTGTGGTCATGAG

Supplementary Table 4. Antisense oligonucleotide (ASO) sequences

ASO	5’ to 3’
Tnik ASO	ATGTATTCCTGAAGAGGCAGCAGCA
Ctrl ASO	CCTCTTACCTCAGTTACAATTTATA

Supplementary Table 5. Differentially spliced genes upon PTBP1 deficiency

Supplementary Table 6. Differentially expressed genes upon PTBP1 deficiency

Supplementary Table 7. RNA-binding proteins affected by PTBP1 deficiency

Supplementary Table 8. Differentially expressed genes associated with top 8 GO terms

Supplementary Table 9. Sertoli cell-specific splicing events affected by PTBP1 deficiency

Supplementary Table 10. Conservation of PTBP1-regulated exons of actin regulators between mice and humans
