# Supplementary material for: PTBP1 mediates Sertoli cell actin cytoskeleton organization by regulating alternative splicing of actin regulators
Source: Nucleic Acids Res. 2024 Oct 7;52(20):12244–61. doi: 10.1093/nar/gkae862 (PMC11551747; doi:10.1093/nar/gkae862)
Supplement: gkae862_Supplemental_Files [file gkae862_supplemental_files.zip › Suppmentary figures.pdf]

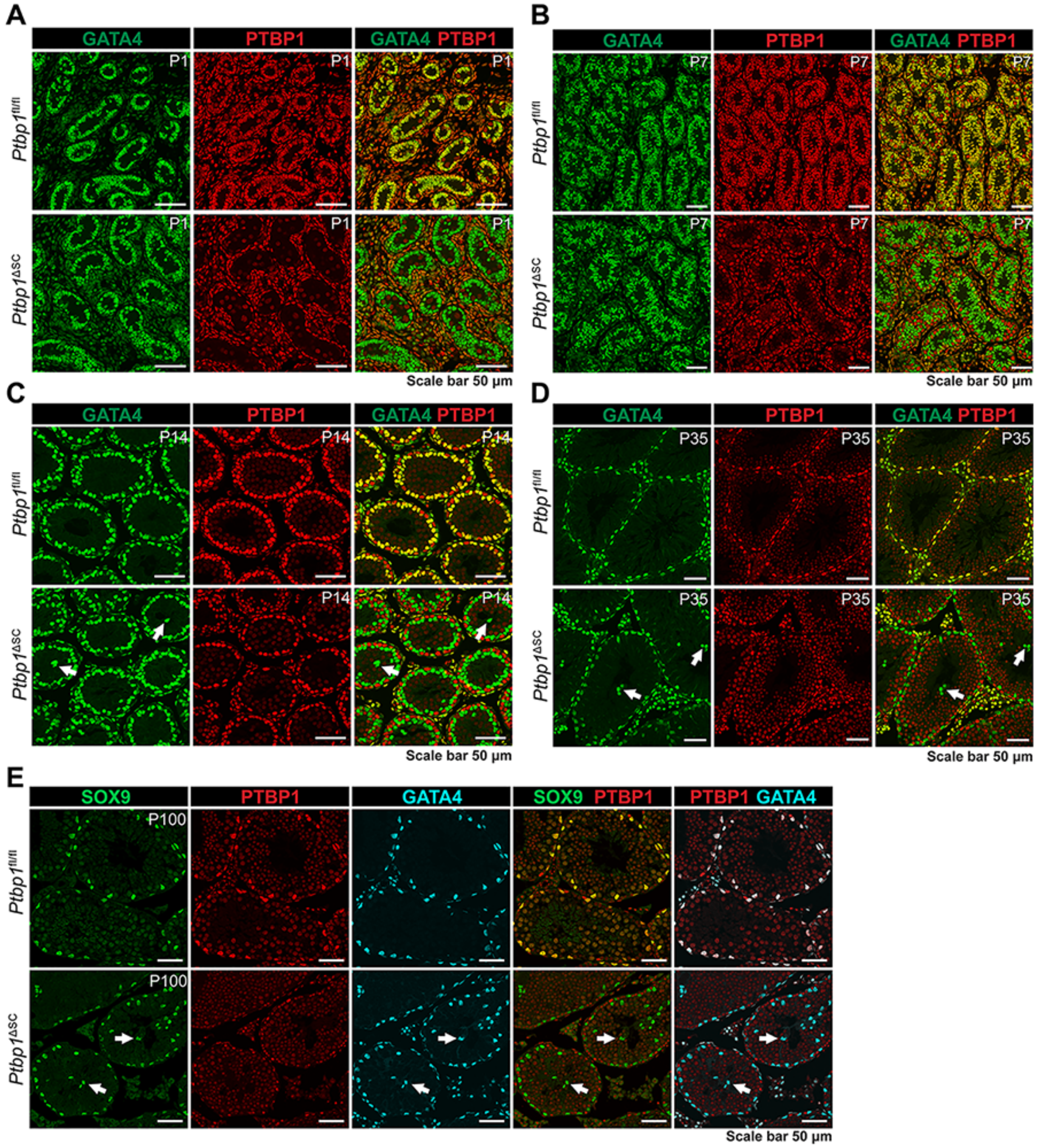

Supplementary Figure S1. PTBP1 was efficiently deleted in Sertoli cells in *Ptbp1*$^{\Delta SC}$ mice

(A-D) Double immunofluorescence staining using the antibodies against PTBP1 and GATA4 shows PTBP1 expression and deletion at the neonatal stage and adulthood. Arrows in C and D point to mislocalized GATA4-positive cell nuclei in the lumens of seminiferous tubules. (E) Triple immunofluorescence staining using the antibodies against PTBP1, GATA4, and SOX9 shows that mislocalized cell nuclei are positive for both GATA4 and SOX9 (arrows), confirming that they are Sertoli cell nuclei.

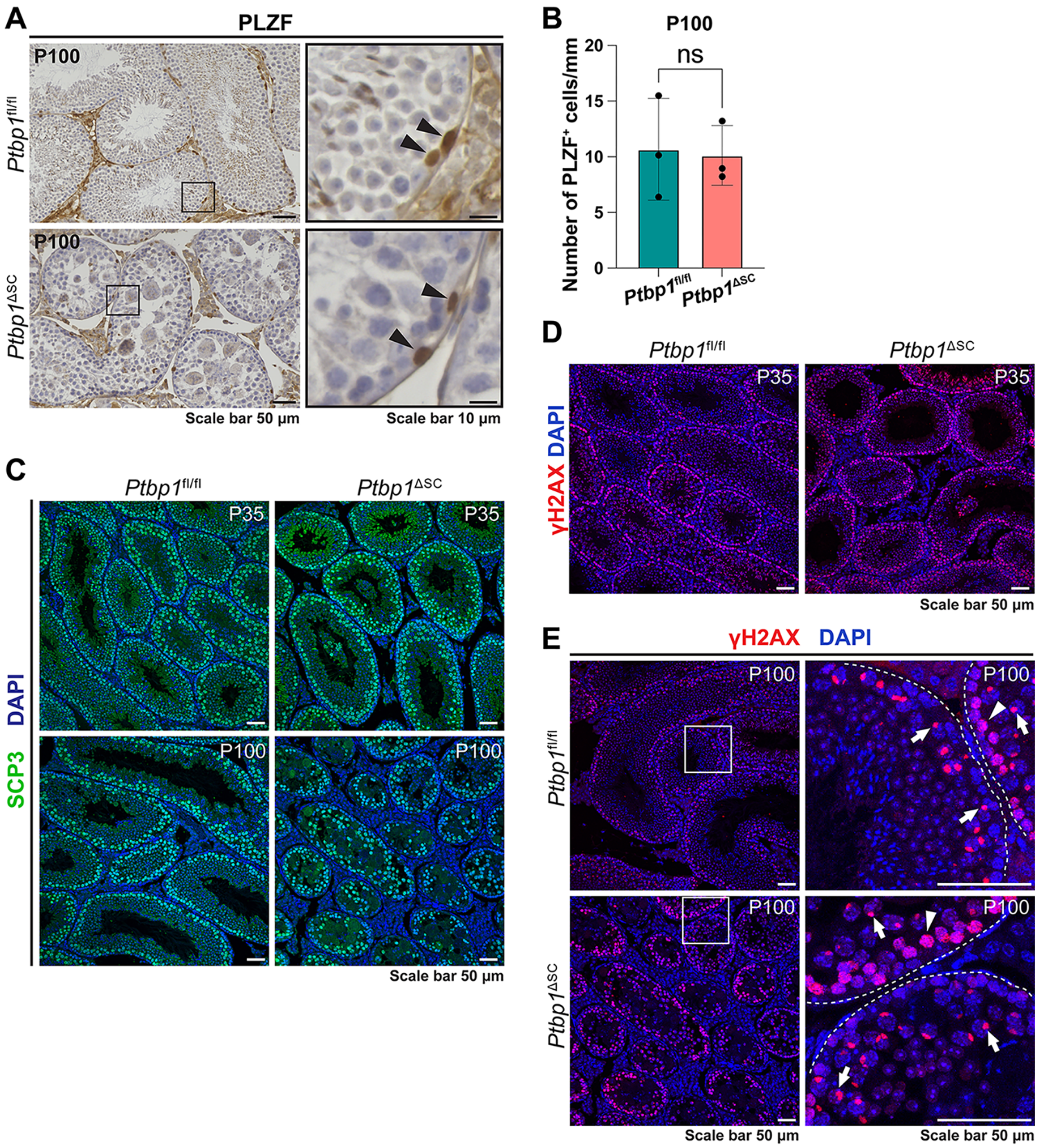

Supplementary Figure S2. Germ cell mitosis and meiosis were not affected by *Ptbp1* deletion (A) Immunohistochemistry staining with an antibody against PLZF shows undifferentiated spermatogonia cells in the testes of *Ptbp1*$^{fl/fl}$ and *Ptbp1*$^{\Delta SC}$ mice at P100. Boxed regions are magnified on the right. Arrowheads indicate PLZF-positive spermatogonia cells. (B) Quantification of PLZF-positive spermatogonia cells. 329 seminiferous tubules from the testis sections of 3 *Ptbp1*$^{fl/fl}$ mice and 390 tubules from 3 *Ptbp1*$^{\Delta SC}$ mice were assessed. The number of PLZF-positive cells in each tubule was divided by the perimeter of the tubule (mm). The resulting values were averaged in each mouse and compared between the two groups. ns, not significant. (C-E) Immunofluorescence staining shows SYCP3-positive spermatocytes or γH2AX-positive germ cells at the leptotene and zygotene stage (arrowheads) in *Ptbp1*$^{fl/fl}$ and *Ptbp1*$^{\Delta SC}$ mice at P35 and P100. Boxed regions were magnified in the right panels in E. γH2AX is condensed in the sex body of germ cells at the pachytene stage (arrows). Dashed lines indicate the edges of seminiferous tubules.

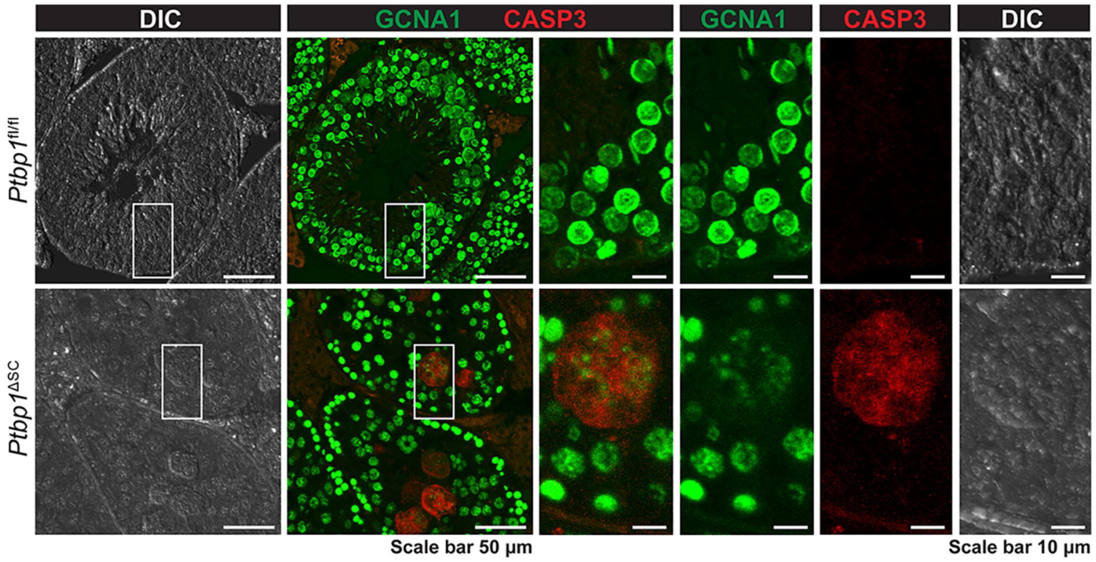

Supplementary Figure S3. Germ cells formed apoptotic multinucleated giant cells in *Ptbp1*$^{\Delta SC}$ mice

Double immunofluorescence staining using the antibodies against GCNA1 and cleaved CASPASE3 (CASP3) shows the multinucleated giant cells formed by germ cells underwent apoptosis at P100. GCNA1 is used for labeling germ cells. Boxed regions are magnified in the right panels. DIC, Differential interference contrast.

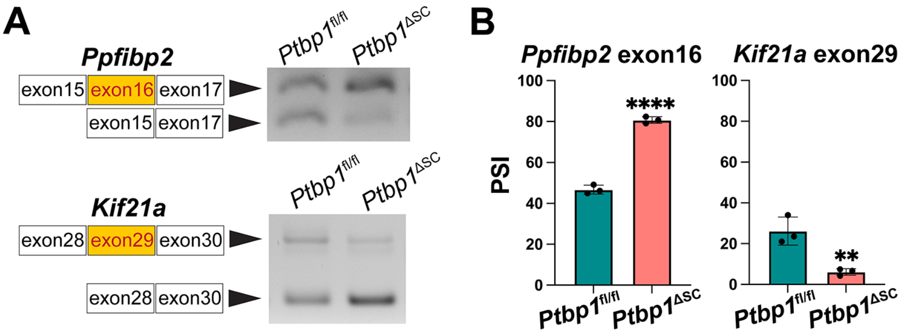

Supplementary Figure S4. Validation of alternatively spliced events in *Ptbp1*$^{\Delta SC}$ mice

(A) Representative gel images show alternatively spliced events in the testes of *Ptbp1*$^{fl/fl}$ and *Ptbp1*$^{\Delta SC}$ mice at P35. (B) shows the quantifications of PSI. 3 *Ptbp1*$^{\Delta SC}$ mice and 3 sibling littermate *Ptbp1*$^{fl/fl}$ mice were assessed. Data are presented as mean± SD. **P<0.01, ****P<0.0001.

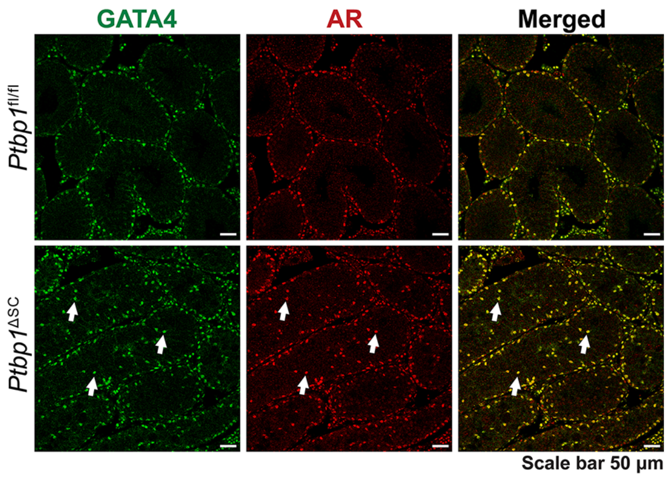

Supplementary Figure S5. Maturation of Sertoli cells is not affected in *Ptbp1*$^{\Delta SC}$ mice

Double immunofluorescence staining using antibodies against GATA4 (to mark Sertoli cells) and androgen receptor (AR) (to mark mature Sertoli cells) shows the maturation of Sertoli cells in *Ptbp1*$^{fl/fl}$ and *Ptbp1*$^{\Delta SC}$ mice at P100. Arrows point to mislocalized Sertoli cell nuclei in *Ptbp1*$^{\Delta SC}$ mice.

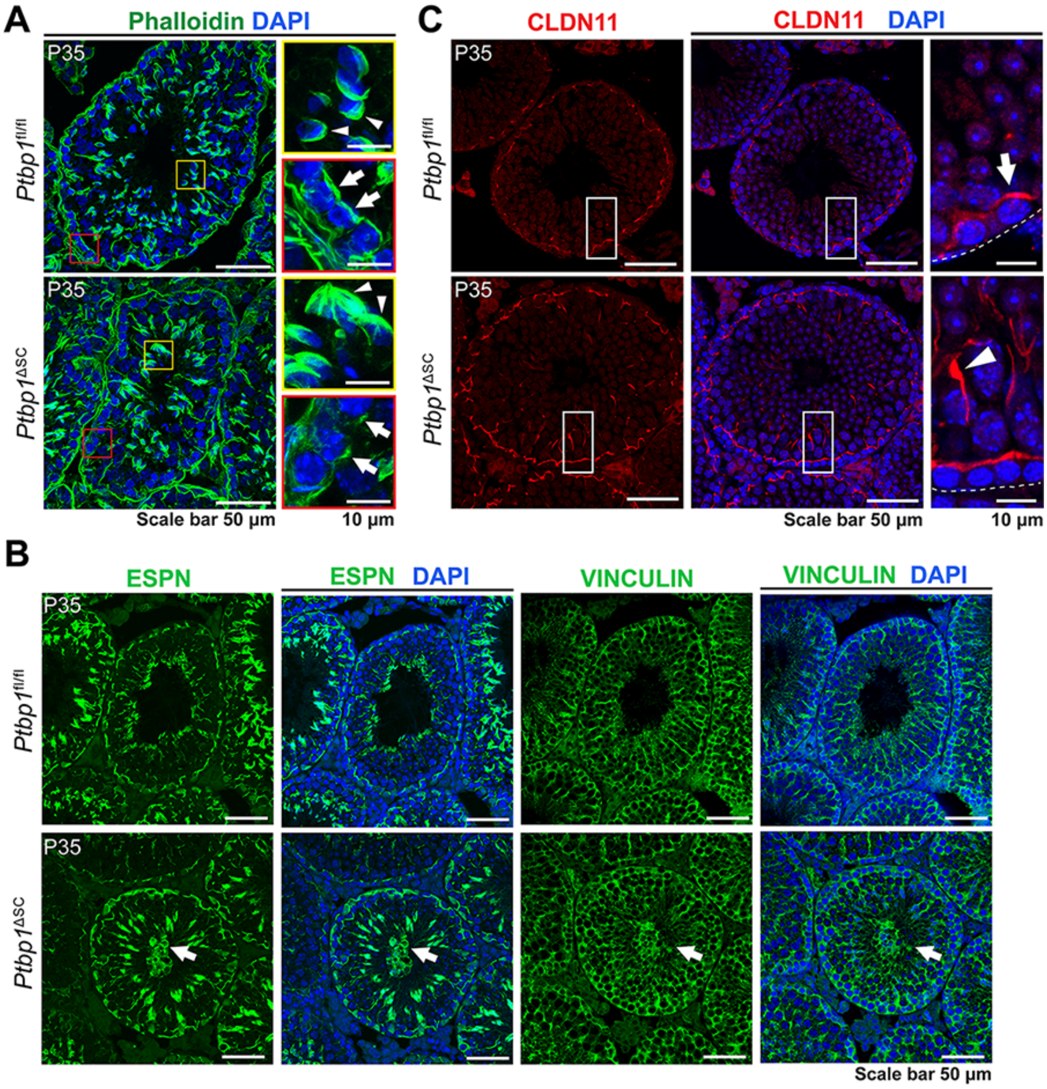

Supplementary Figure S6. ***Ptbp1*$^{\Delta SC}$ mice had disorganized F-actin and mislocalized ES and tight junction protein at P35**

(A) Phalloidin staining shows F-actin organization. The apical ES (yellow boxes) and basal ES (red boxes) regions are magnified in insets. Sertoli cells of *Ptbp1*$^{\Delta SC}$ mice exhibit abnormal actin assembly at the basal ES (arrows) and apical ES (arrowheads). (B) Immunofluorescence staining using the antibodies against ESPN and VINCULIN on the series testis sections shows the mislocalization of ESPN and VINCULIN, at P35. Arrows pointed to mislocalized ESPN and VINCULIN in the lumen. (C) Immunofluorescence staining using the antibody against CLDN11 shows the misdistribution of tight junction protein in the *Ptbp1*$^{\Delta SC}$ testis. Boxed regions are magnified on the right. The arrow shows the normal distribution of CLDN11 at the basal region of Sertoli cells in *Ptbp1*$^{fl/fl}$ mice. The arrowhead shows mislocalized CLDN11 in *Ptbp1*$^{\Delta SC}$ mice.

TNIK protein sequence alignment

```
# Length: 1360
# Identity:    1344/1360 (98.8%)
# Similarity:  1355/1360 (99.6%)
# Gaps:           0/1360 (0.0%)

Mouse_TNIK      1 MASDSPARSLDEIDLSALRDPAGIFELVELVGNGTYGQVYKGRHVKTGQL     50
                  ||||||||||||||||||||||||||||||||||||||||||||||||||
Human_TNIK      1 MASDSPARSLDEIDLSALRDPAGIFELVELVGNGTYGQVYKGRHVKTGQL     50

Mouse_TNIK     51 AAIKVMDVTGDEEEEIKQEINMLKKYSHHRNIATYYGAFIKKNPPGMDDQ    100
                  ||||||||||||||||||||||||||||||||||||||||||||||||||
Human_TNIK     51 AAIKVMDVTGDEEEEIKQEINMLKKYSHHRNIATYYGAFIKKNPPGMDDQ    100

Mouse_TNIK    101 LWLVMEFCGAGSVTDLIKNTKGNTLKEEWIAYICREILRGLSHLHQHKVI    150
                  ||||||||||||||||||||||||||||||||||||||||||||||||||
Human_TNIK    101 LWLVMEFCGAGSVTDLIKNTKGNTLKEEWIAYICREILRGLSHLHQHKVI    150

Mouse_TNIK    151 HRDIKGQNVLLTENAEVKLVDFGVSAQLDRTVGRRNTFIGTPYWMAPEVI    200
                  ||||||||||||||||||||||||||||||||||||||||||||||||||
Human_TNIK    151 HRDIKGQNVLLTENAEVKLVDFGVSAQLDRTVGRRNTFIGTPYWMAPEVI    200

Mouse_TNIK    201 ACDENPDATYDFKSDLWSLGITAIEMAEGAPPLCDMHPMRALFLIPRNPA    250
                  ||||||||||||||||||||||||||||||||||||||||||||||||||
Human_TNIK    201 ACDENPDATYDFKSDLWSLGITAIEMAEGAPPLCDMHPMRALFLIPRNPA    250

Mouse_TNIK    251 PRLKSKKWSKKFQSFIESCLVKNHSQRPATEQLMKHPFIRDQPNERQVRI    300
                  ||||||||||||||||||||||||||||||||||||||||||||||||||
Human_TNIK    251 PRLKSKKWSKKFQSFIESCLVKNHSQRPATEQLMKHPFIRDQPNERQVRI    300

Mouse_TNIK    301 QLKDHIDRTKKKRGEKDETEYEYSGSEEEEEENDSGEPSSILNLPGESTL    350
                  ||||||||||||||||||||||||||||||||||||||||||||||||||
Human_TNIK    301 QLKDHIDRTKKKRGEKDETEYEYSGSEEEEEENDSGEPSSILNLPGESTL    350

Mouse_TNIK    351 RRDFLRLQLANKERSEALRRQQLEQQQRENEEHKRQLLAERQKRIEEQKE    400
                  ||||||||||||||||||||||||||||||||||||||||||||||||||
Human_TNIK    351 RRDFLRLQLANKERSEALRRQQLEQQQRENEEHKRQLLAERQKRIEEQKE    400

Mouse_TNIK    401 QRRRLEEQQRREKELRKQQEREQRRHYEEQMRREEERRRAEHEQEYIRRQ    450
                  ||||||||||||||||||||||||||||||||||||||||||||||||||
Human_TNIK    401 QRRRLEEQQRREKELRKQQEREQRRHYEEQMRREEERRRAEHEQEYIRRQ    450

Mouse_TNIK    451 LEEEQRQLEILQQQLLHEQALLLEYKRKQLEEQRQAERLQRQLKQERDYL    500
                  ||||||||||||||||||||||||||||||||||||||||||||||||||
Human_TNIK    451 LEEEQRQLEILQQQLLHEQALLLEYKRKQLEEQRQAERLQRQLKQERDYL    500

Mouse_TNIK    501 VSLQHQRQEQRPLEKKPLYHYKEGMSPSEKPAWAKEVEERSRLNRQSSPA    550
                  ||||||||||||:|||||||||||||||||||||||||||||||||||||
Human_TNIK    501 VSLQHQRQEQRPVEKKPLYHYKEGMSPSEKPAWAKEVEERSRLNRQSSPA    550

Mouse_TNIK    551 MPHKVANRISDPNLPPRSESFSISGVQPARTPPMLRPVDPQIPQLVAVKS    600
                  |||||||||||||||||||||||||||||||||||||||||||.||||||
Human_TNIK    551 MPHKVANRISDPNLPPRSESFSISGVQPARTPPMLRPVDPQIPHLVAVKS    600

Mouse_TNIK    601 QGPALTASQSVHEQPTKGLSGFQEALNVTSHRVEMPRQNSDPTSENPPLP    650
                  ||||||||||||||||||||||||||||||||||||||||||||||||||
Human_TNIK    601 QGPALTASQSVHEQPTKGLSGFQEALNVTSHRVEMPRQNSDPTSENPPLP    650

Mouse_TNIK    651 TRIEKFDRSSWLRQEEDIPPKVPQRTTSISPALARKNSPGNGSALGPRLG    700
                  ||||||||||||||||||||||||||||||||||||||||||||||||||
Human_TNIK    651 TRIEKFDRSSWLRQEEDIPPKVPQRTTSISPALARKNSPGNGSALGPRLG    700

Mouse_TNIK    701 SQPIRASNPDLRRTEPVLESSLQRTSSGSSSSSSTPSSQPSSQGGSQPGS    750
                  ||||||||||||||||:|||.|||||||||||||||||||||||||||||
Human_TNIK    701 SQPIRASNPDLRRTEPILESPLQRTSSGSSSSSSTPSSQPSSQGGSQPGS    750

Mouse_TNIK    751 QAGSSERSRVRANSKSEGSPVLPHEPSKVKPEESRDITRPSRPASYKKAI    800
                  |||||||:||||||||||||||||||:|||||||||||||||||||||||
Human_TNIK    751 QAGSSERTRVRANSKSEGSPVLPHEPAKVKPEESRDITRPSRPASYKKAI    800

Mouse_TNIK    801 DEDLTALAKELRELRIEETNRPLKKVTDYSSSSEESESSEEEEEDGESET    850
                  ||||||||||||||||||||||:|||||||||||||||||||||||||||
Human_TNIK    801 DEDLTALAKELRELRIEETNRPMKKVTDYSSSSEESESSEEEEEDGESET    850

Mouse_TNIK    851 HDGTVAVSDIPRLIPTGAPGNNEQYNMGMVGTHGLETSHADTFGGSISRE    900
                  ||||||||||||||||||||:|||||:||||||||||||||:|.||||||
Human_TNIK    851 HDGTVAVSDIPRLIPTGAPGSNEQYNVGMVGTHGLETSHADSFSGSISRE    900

Mouse_TNIK    901 GTLMIRETAEEKKRSGHSDSNGFAGHINLPDLVQQSHSPAGTPTEGLGRV    950
                  ||||||||:.||||||||||||||||||||||||||||||||||||||||
Human_TNIK    901 GTLMIRETSGEKKRSGHSDSNGFAGHINLPDLVQQSHSPAGTPTEGLGRV    950

Mouse_TNIK    951 STHSQEMDSGAEYGIGSSTKASFTPFVDPRVYQTSPTDEDEEDDESSAAA   1000
                  ||||||||||.|||:||||||||||||||||||||||||||||||:||||||
Human_TNIK    951 STHSQEMDSGTEYGMGSSTKASFTPFVDPRVYQTSPTDEDEEDEESSAAA   1000

Mouse_TNIK   1001 LFTSELLRQEQAKLNEARKISVVNVNPTNIRPHSDTPEIRKYKKRFNSEI   1050
                  ||||||||||||||||||||||||||||||||||||||||||||||||||
Human_TNIK   1001 LFTSELLRQEQAKLNEARKISVVNVNPTNIRPHSDTPEIRKYKKRFNSEI   1050

Mouse_TNIK   1051 LCAALWGVNLLVGTENGLMLLDRSGQGKVYNLINRRRFQQMDVLEGLNVL   1100
                  ||||||||||||||||||||||||||||||||||||||||||||||||||
Human_TNIK   1051 LCAALWGVNLLVGTENGLMLLDRSGQGKVYNLINRRRFQQMDVLEGLNVL   1100

Mouse_TNIK   1101 VTISGKKNKLRVYYLSWLRNRILHNDPEVEKKQGWITVGDLEGCIHYKVV   1150
                  ||||||||||||||||||||||||||||||||||||||||||||||||||
Human_TNIK   1101 VTISGKKNKLRVYYLSWLRNRILHNDPEVEKKQGWITVGDLEGCIHYKVV   1150

Mouse_TNIK   1151 KYERIKFLVIALKNAVEIYAWAPKPYHKFMAFKSFADLQHKPLLVDLTVE   1200
                  ||||||||||||||||||||||||||||||||||||||||||||||||||
Human_TNIK   1151 KYERIKFLVIALKNAVEIYAWAPKPYHKFMAFKSFADLQHKPLLVDLTVE   1200

Mouse_TNIK   1201 EGQRLKVIFGSHTGFHVIDVDSGNSYDIYIPSHIQGNITPHAIVILPKTD   1250
                  ||||||||||||||||||||||||||||||||||||||||||||||||||
Human_TNIK   1201 EGQRLKVIFGSHTGFHVIDVDSGNSYDIYIPSHIQGNITPHAIVILPKTD   1250

Mouse_TNIK   1251 GMEMLVCYEDEGVYVNTYGRITKDVVLQWGEMPTSVAYIHSNQIMGWGEK   1300
                  ||||||||||||||||||||||||||||||||||||||||||||||||||
Human_TNIK   1251 GMEMLVCYEDEGVYVNTYGRITKDVVLQWGEMPTSVAYIHSNQIMGWGEK   1300

Mouse_TNIK   1301 AIEIRSVETGHLDGVFMHKRAQRLKFLCERNDKVFFASVRSGGSSQVFFM   1350
                  ||||||||||||||||||||||||||||||||||||||||||||||||||
Human_TNIK   1301 AIEIRSVETGHLDGVFMHKRAQRLKFLCERNDKVFFASVRSGGSSQVFFM   1350

Mouse_TNIK   1351 TLNRNSMMNW   1360
                  ||||||||||
Human_TNIK   1351 TLNRNSMMNW   1360
```

Supplementary Figure S7. Alignment of Mouse and human TNIK protein sequences

Mouse and human TNIK proteins share a 99% similarity. Exon 14 encoded 29 amino acids are 100% identical (indicated by the red line above the sequence) between human and mouse TNIK. The green line above the sequence indicates exon 21 encoded amino acids.

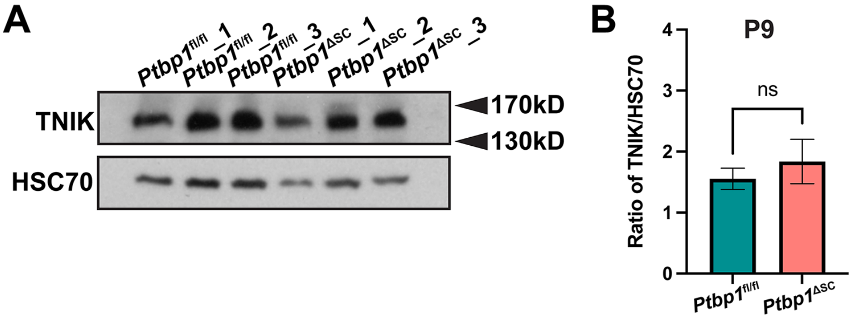

Supplementary Figure S8. PTBP1 deficiency does not alter the protein level of TNIK
(A-B) Western blotting result shows the total levels of TNIK protein in the testes of *Ptbp1*$^{fl/fl}$ and *Ptbp1*$^{\Delta SC}$ mice at P9 are comparable. The quantification of the ratio of TNIK to HSC70 in the testes is shown in B. Mice used in the assay are littermates. Data are presented as mean± SD. ns, not significant.

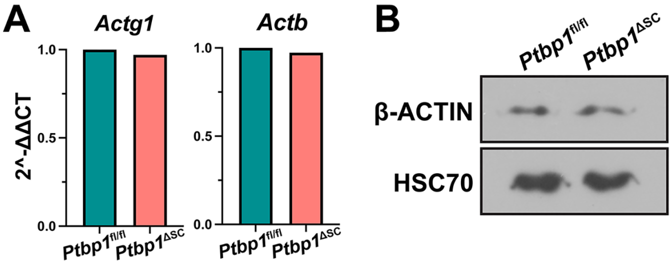

Supplementary Figure S9. The amount of actin is not decreased in PTBP1-deficient Sertoli cells
(A) Quantitative real-time PCR result shows the mRNA levels of non-muscle β-ACTIN and β-ACTIN in cultured primary Sertoli cells from *Ptbp1*$^{fl/fl}$ and *Ptbp1*$^{\Delta SC}$ mice are comparable. (B) Western blotting result shows the protein level of β-ACTIN in cultured primary Sertoli cells from *Ptbp1*$^{fl/fl}$ and *Ptbp1*$^{\Delta SC}$ mice is also comparable.

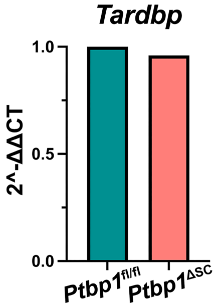

Supplementary Figure S10. PTBP1 deficiency does not alter the expression of *Tardbp*
Quantitative real-time PCR result shows that PTBP1 deficiency does not alter the mRNA level of *Tardbp* in cultured primary Sertoli cells. *Tardbp* encodes the RNA-binding protein TDP-43.

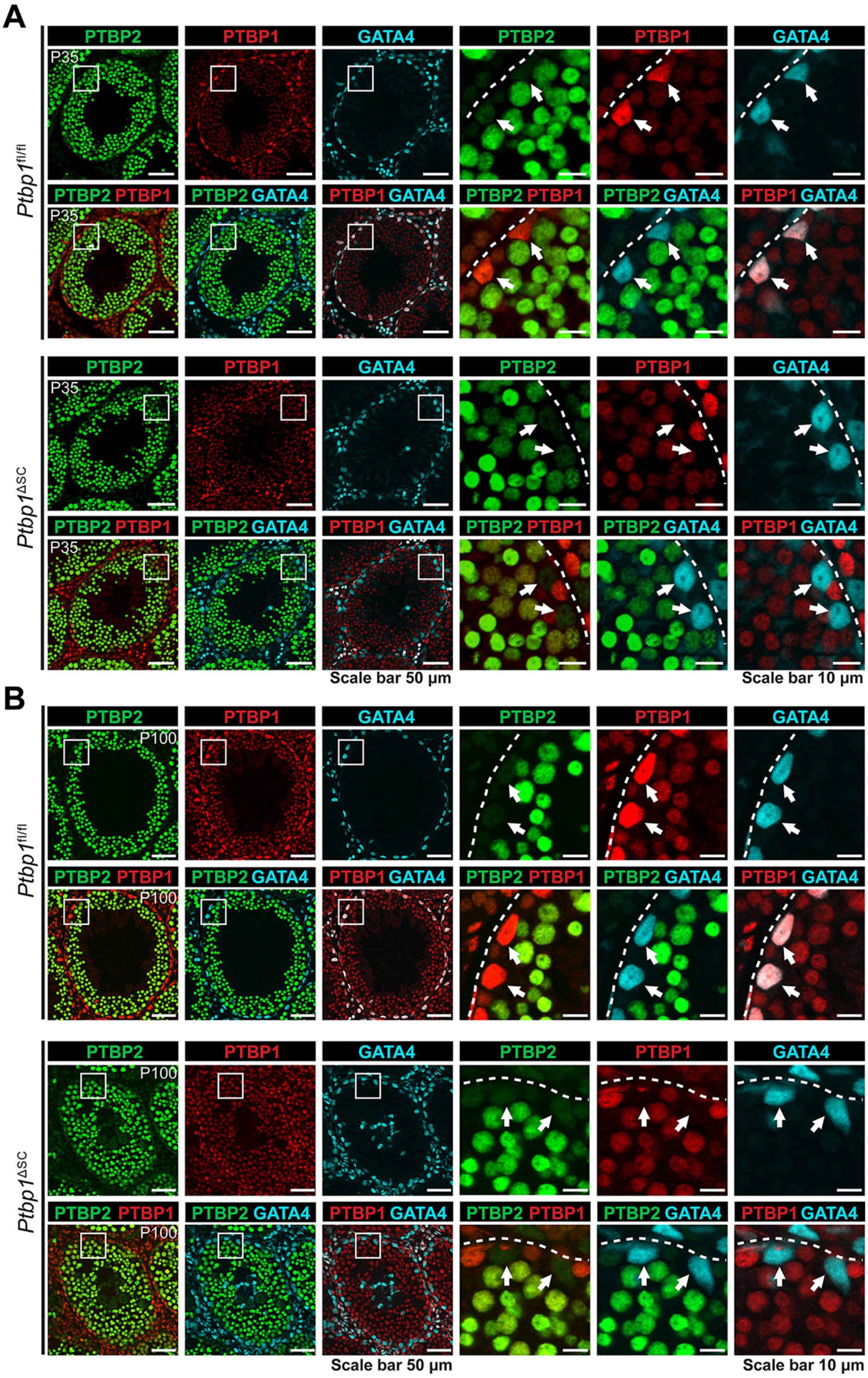

Supplementary Figure S11. PTBP1 deficiency does not increase PTBP2 expression in Sertoli cells

Triple immunofluorescence staining using the antibodies against PTBP1, PTBP2, and GATA4 shows PTBP1 and PTBP2 expression in testes at P35 and P100. Arrows point to Sertoli cells positive for PTBP1 but negative for PTBP2 in *Ptbp1*$^{fl/fl}$ mice, or Sertoli cells negative for both PTBP1 and PTBP2 in *Ptbp1*$^{\Delta SC}$ mice. The white dotted lines show the edges of the seminiferous tubules. Boxed regions in the left three panels were magnified in the right three.
